# A randomized study to compare oral potassium binders in the treatment of acute hyperkalemia

**DOI:** 10.1186/s12882-023-03145-x

**Published:** 2023-04-05

**Authors:** Alejandro E. Cañas, Hayden R. Troutt, Luohua Jiang, Sam Tonthat, Omar Darwish, Antoney Ferrey, Shahram Lotfipour, Kamyar Kalantar-Zadeh, Ramy Hanna, Wei Ling Lau

**Affiliations:** 1 Division of Nephrology, Department of Medicine, University of California-Irvine, 101 The City Drive South, City Tower, Suite 400, Orange, CA 92868 USA; 2 Department of Epidemiology & Biostatistics, Program in Public Health, University of California-Irvine, Irvine, USA; 3 Department of Medicine, University of California-Irvine, Orange, USA; 4 Department of Emergency Medicine, University of California-Irvine, Orange, USA

**Keywords:** Acute hyperkalemia, Cation-exchange resins, Sodium polystyrene sulfonate, Patiromer, Sodium zirconium cyclosilicate

## Abstract

**Background:**

The KBindER (K^+^ Binders in Emergency Room and hospitalized patients) clinical trial is the first head-to-head evaluation of oral potassium binders (cation-exchange resins) for acute hyperkalemia therapy.

**Methods:**

Emergency room and hospitalized patients with a blood potassium level ≥ 5.5 mEq/L are randomized to one of four study groups: potassium binder drug (sodium polystyrene sulfonate, patiromer, or sodium zirconium cyclosilicate) or nonspecific laxative (polyethylene glycol). Exclusion criteria include recent bowel surgery, ileus, diabetic ketoacidosis, or anticipated dialysis treatment within 4 h of treatment drug. Primary endpoints include change in potassium level at 2 and 4 h after treatment drug. Length of hospital stay, next-morning potassium level, gastrointestinal side effects and palatability will also be analyzed. We are aiming for a final cohort of 80 patients with complete data endpoints (20 per group) for comparative statistics including multivariate adjustment for kidney function, diabetes mellitus, congestive heart failure, metabolic acidosis, renin-angiotensin-aldosterone system inhibitor prescription, and treatment with other agents to lower potassium (insulin, albuterol, loop diuretics).

**Discussion:**

The findings from our study will inform decision-making guidelines on the role of oral potassium binders in the treatment of acute hyperkalemia.

**Trial registration:**

ClinicalTrials.gov Identifier: NCT04585542. Registered 14 October 2020.

**Supplementary Information:**

The online version contains supplementary material available at 10.1186/s12882-023-03145-x.

## Introduction

In the setting of normal kidney function, serum potassium (K^+^) is maintained within a tight range (3.5–5.3 mEq/L) via renal excretion and cellular redistribution [1]. Hyperkalemia is considered severe if K^+^ is ≥ 6.0 mEq/L, with or without symptomatic muscle weakness/paralysis or electrocardiogram changes. Severe hyperkalemia occurs in up to 10% of all hospitalized patients [2] and is an independent predictor of increased mortality [3] that is associated with prolonged hospital stays, higher healthcare costs, and a 30-day hospital readmission rate of 14.21% vs. 9.86% in non-hyperkalemic patients [4].

Risk factors for hyperkalemia include chronic kidney disease (CKD), diabetes mellitus [5–7], and use of certain medications such as renin-angiotensin-aldosterone system (RAAS) inhibitors and mineralocorticoid receptor antagonists [5, 8–10]. Historically, discontinuation of RAAS inhibitors occurred in ~75% of cases in response to drug-induced hyperkalemia [11], potentially leaving patients vulnerable to adverse cardiovascular events [12]. The emergence of the newer oral potassium binders (patiromer and sodium zirconium cyclosilicate [SZC]) has allowed maintenance of normokalemia in CKD patients receiving background therapy with RAAS inhibitors [13–15]. However, there is a lack of data on the potential utility of these binders for the treatment of acute hyperkalemia.

No universally accepted standard of care protocol has been established for the treatment of patients presenting with acute hyperkalemia, whether in the ambulatory or emergency room (ER) setting [2, 11, 16]. In a multicenter, prospective, observational analysis (*n* = 203), the REVEAL-ED study reported that 7 therapies (inhaled β2-agonists, intravenous [i.v.] bicarbonate, i.v. calcium, hemodialysis, i.v. diuretics, i.v. insulin/glucose, oral sodium polystyrene sulfonate) were utilized in 43 varying combinations in the 14 emergency departments surveyed [17].

**Fig. 1:**
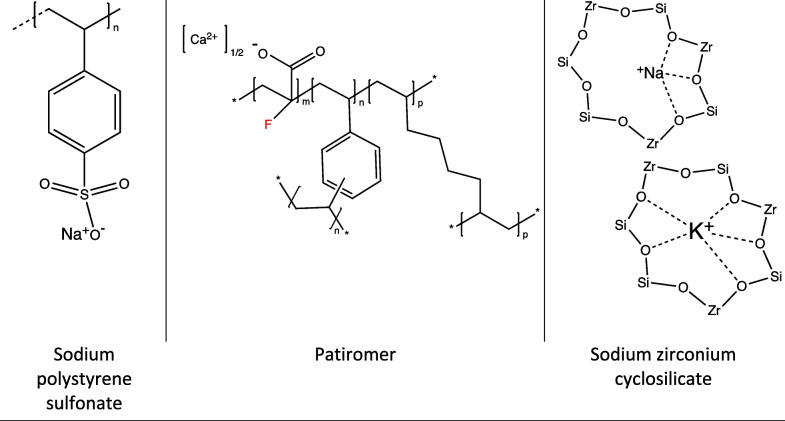
Chemical structure of the oral potassium binders (cation-exchange resins). Sodium zirconium cyclosilicate has a conformational change when sodium (Na^+^) is exchanged for potassium (K^+^)

Oral potassium binders hold particular appeal in patients with advanced CKD where diuretics may be less effective, and where dialysis has inherent risks and is costly. For over 50 years the only available oral potassium binder had been sodium polystyrene sulfonate (SPS, FDA approved in 1958). In recent years, two new agents have been approved by the FDA for chronic management of hyperkalemia (patiromer in 2015 and SZC in 2018) (Fig. 1). Clinical trials where oral potassium binders were studied against placebo are summarized in Table 1. To date, no studies have compared the efficacy of SPS, patiromer and SZC head-to-head in ER or hospitalized patients with acute hyperkalemia.

**Table 1:** Summary of clinical trials with oral potassium binders

**Clinical trial**	**Number of participants & dosage**	**Study population**	**Study type**	**Duration**	**Primary endpoints**	**Acute serum K**^**+**^** findings**	**Limitations**
**Sodium polystyrene sulfonate**							
Randomized Clinical Trial of Sodium Polystyrene Sulfonate for the Treatment of Mild Hyperkalemia in CKD [18] Lepage et al.	33 patients total; 16 assigned to SPS treatment group (30 g/day) and 17 assigned to placebo group	Adult patients in ambulatory setting with chronic kidney disease and baseline serum K^+^ of 5.0–5.9 mEq/L	Double-blind, randomized clinical controlled trial	7 days	Comparison of serum K^+^ between groups from baseline to day 7 (after final treatment dose)	Decreased serum K^+^ levels by a mean of 1.25 mEq/L (SD: ± 0.56) in SPS group compared to 0.21 mEq/L (SD: ± 0.29) in placebo group over 7-day period; difference of -1.04 mEq/L (95% CI, -1.37 to -0.71); *P* < 0.001	Small sample size; serum K^+^ of > 5.9 mEq/L was excluded; limited to 2 blood draws for serum K^+^ (day 0 and day 7) hence, unable to detect efficacy in acute setting
**Patiromer**							
Patiromer for Treatment of Hyperkalemia in the Emergency Department: A Pilot Study [19] Rafique et al.	30 patients total; 15 assigned to standard of care group, 15 assigned to patiromer group receiving single, 25.2 g dose	Adult emergency room patients with end-stage renal disease and baseline serum K^+^ ≥ 6.0 mEq/L	Single-center, randomized, open-label pilot study	6 h	Difference in serum K^+^ between SOC group and treatment group at 6 h	Decreased serum K^+^ within 2 h of the patiromer-treated group (single-dose 25.2 g) compared to the SOC group (5.91 mEq/L vs. 6.51 mEq/L respectively; *P* < 0.009); no differences shown at 6 h	Small sample size
Patiromer Induces Rapid and Sustained Potassium Lowering in Patients with Chronic Kidney Disease and Hyperkalemia [13] Bushinsky et al.	25 patients in single treatment group receiving 8.4 g/dose, 4 doses total	Adult patients hospitalized with serum K^+^ of 5.5–6.5 mEq/L; majority with chronic kidney disease and all taking at least one RAAS inhibitor	Multicenter, international, phase I, open-label, single-arm study conducted at 5 sites in Europe	72-h K^+^/Na^+^ restricted diet phase followed by 48-h treatment phase and outpatient follow-up phase	Mean change in serum K^+^ from baseline until 48 h after initial dose	Decreased serum K^+^ of 0.21 mEq/L within 7 h of initial treatment compared to baseline (95% CI, -0.35 to -0.07) P = 0.004. Significant reductions occurred at all assessment points from 7–48 h *P* ≤ 0.004 at 10 h, *P* ≤ 0.001 at 12–48 h	Lack of comparative placebo control and small sample size. Those with abnormal electrocardiograph readings related to hyperkalemia were excluded
**Sodium zirconium cyclosilicate**							
Emergency Potassium Normalization Treatment Including Sodium Zirconium Cyclosilicate: A Phase II, Randomized, Double-blind, Placebo-controlled Study (ENERGIZE) [16] Peacock et al.	62 patients total; 29 assigned to SZC group (10 g, up to 3 doses); 33 assigned to placebo group	Adult patients admitted to the emergency room with baseline serum K^+^ of ≥ 5.8 mEq/L	Multicenter, international, randomized, double-blind, placebo-controlled, parallel-group, phase II study conducted at 33 sites in Denmark, Italy, Russia, and the United States	10-h treatment period followed by a single follow-up visit 7 days later	Mean change in serum K^+^ from baseline until 4 h after initial dose	Greater reduction in baseline serum K^+^ within SZC treatment group compared to placebo group –0.72 (0.12) versus –0.36 (0.11) mEq/L (least squares mean difference –0.35 mmol/L; 95% CI = –0.68 to –0.02)	Missing lab values of serum K^+^ at 4 h for substantial amount of patients; small sample size of patients (*N* = 62); enrolled; high withdrawal rate (30.2% treatment group and 43.2% placebo group)
Sodium Zirconium Cyclosilicate in Hyperkalemia [20] Packham et al.	754 patients assigned to 4 different SZC treatment groups (1.5 g, 2.5 g, 5 g, 10 g) or placebo group	Adult patients in the ambulatory setting with baseline serum K^+^ of 5.0 – 6.5 mEq/L	Multicenter, two-stage, randomized, double-blind, phase III study conducted at 65 sites in United States, Australia, and South Africa	Initial phase of 48 h, followed by a 12-day maintenance phase	Exponential rate of change in mean serum K^+^ at 48 h and between-group difference of serum K^+^ post 48 h during maintenance phase	Mean reduction of serum K^+^ with single dose of 10 g SZC within 1 h was 0.11 mEq/L compared to 0.01 mEq/L in placebo group (95% CI, -0.05 to 0.07)	Patients with serum K^+^ of > 6.5 mEq/L and those with abnormal electrocardiograph readings related to hyperkalemia were excluded; hospitalized patients were excluded
Effect of Sodium Zirconium Cyclosilicate on Potassium Lowering for 28 Days Among Outpatients with Hyperkalemia – the HARMONIZE Randomized Clinical Trial [21] Kosiborod et al.	258 patients assigned to 10 g SZC treatment group (open-label phase) followed by randomization to 3 different SZC dosage groups (5, 10, or 15 g) or placebo (28-day phase)	Adult patients in the ambulatory setting with baseline serum K^+^ of ≥ 5.1 mEq/L	Phase III, randomized, double-blind, placebo-controlled trial conducted at 44 sites in United States, Australia, and South Africa	Initial open-label phase of 48 h, followed by a randomized 28-day phase	Difference of mean serum K^+^ levels between 3 different SZC treatment groups and placebo group	Decreased serum K^+^ of 0.2 mEq/L within 1 h of single 10 g dose of SZC compared to baseline (95% CI, -0.3 to -0.2) *P* < 0.001. At 2 and 4 h after initial dose, mean change in serum K^+^ was -0.4 mEq/L (95% CI, -0.5 to -0.4 and -0.5 mEq/L (95% CI, -0.6 to -0.5); *P* < 0.001 for each time point	Hospitalized patients and those with severe arrythmias were excluded
A Phase 2 Study on the Treatment of Hyperkalemia in Patients with Chronic Kidney Disease Suggests that the Selective Potassium Trap, ZS-9, is Safe and Efficient [22] Ash et al.	90 patients total; 60 assigned to 3 different SZC dosage groups (0.3, 3, or 10 g); 30 assigned to placebo group	Adult patients with stage 3 chronic kidney disease and baseline serum K^+^ of 5.0–6.0 mEq/L	Phase II, randomized, double-blind, placebo-controlled trial conducted at 9 sites in the United States	48 h	Rate of serum K^+^ decline within the first 48 h	Decreased serum K^+^ of 0.11 mEq/L from baseline at 1 h following initial dose in 10 g SZC group compared to placebo (*P* = 0.02)	Small sample size of patients; serum K^+^ of > 6.0 mEq/L was excluded

### Sodium polystyrene sulfonate (SPS)

SPS was introduced to the market in 1958 prior to the Kefauver-Harris Drug Amendments, laws that established protocols for drug safety and efficacy [18]. SPS can be administered orally or rectally, and exchanges Na^+^ for K^+^ within the distal colon. The drug also has an affinity for divalent cations including calcium (Ca^2+^) and magnesium (Mg^2+^), thus there is a potential risk of hypocalcemia and hypomagnesemia. In rare cases, SPS has been associated with intestinal necrosis and increased mortality when combined with 70% sorbitol, resulting in a 2009 FDA black-box warning against long-term use for hyperkalemia management [23, 24]. Sorbitol was initially added for its laxative properties, to minimize bowel impaction with SPS. A review of case reports published between 1948–2011 identified 58 cases of SPS serious adverse events (41 preparations with sorbitol and 17 preparations without sorbitol); there was an associated 33% mortality rate [23]. This is a rare event; approximately 5 million doses of SPS are administered in the United States annually [25].

SPS was the only K^+^ lowering resin available for over 50 years, and remains widely used among clinicians today (without sorbitol or with much lower concentrations of the sugar-alcohol) [23]. There has been one randomized controlled trial which compared SPS vs. placebo in the outpatient setting, in 31 CKD patients with mild hyperkalemia (5.0–5.9 mEq/L). SPS without sorbitol dosed 30 g/day for 7 days was effective in decreasing serum K^+^ by an average of 1.04 mEq/L [18]. While SPS is widely used as an adjunct therapy for acute hyperkalemia, randomized controlled trials in the ER and hospital setting are lacking.

### Patiromer

Patiromer was FDA-approved in 2015 and has been tested extensively in the management of chronic hyperkalemia [13, 14, 26, 27]. Patiromer is a non-absorbable spherical polymer that exchanges Ca^2+^ for K^+^ in the distal colon. In vitro animal studies conducted by Lingyun et al. demonstrated that patiromer had a 1.5–2.5-fold superior K^+^ binding capacity compared to SPS under colonic-like pH conditions [28]. Ca^2+^ loading in CKD patients may contribute to vascular calcification. However, in the 52-week study conducted by Bakris et al. (*n* = 304), there was no increase in serum Ca^2+^ amongst patients treated daily with patiromer [27]. Similar to SPS, binding of Mg^2+^ can occur and cases of patiromer-associated hypomagnesemia have been reported in CKD patients [29].

The OPAL-HK [14], PEARL-HF [30] and AMETHYST-DN [27] clinical trials demonstrated efficacy of patiromer for chronic management of hyperkalemia, in CKD patients with/without comorbidities such as heart failure, diabetes mellitus, and hypertension. Importantly, these studies showed that patiromer allowed safe continuation of RAAS inhibitor therapy with avoidance of drug-induced hyperkalemia.

To date, only one randomized controlled trial has investigated patiromer in the setting of acute hyperkalemia, in end-stage kidney disease patients. Rafique et al. reported significant decrease in serum K^+^ 2 h after patiromer therapy (*n* = 15 per group, baseline average serum K^+^ 6.32 mEq/L, follow up serum K^+^ of 5.91 mEq/L vs. 6.51 mEq/L in the standard of care group; *P* < 0.009) [19].

### Sodium zirconium cyclosilicate (SZC)

FDA-approved in 2018, SZC binds K^+^ in exchange for sodium and hydrogen ions within the small intestine. SZC mimics physiological K^+^ ion channels and has over 25-fold selectivity for K^+^ over other ions such as Ca^2+^ and Mg^2+^, in comparison to SPS which shows a 0.2–0.3 selectivity for K^+^ over these ions [31]. The safety and efficacy of SZC for the management of chronic hyperkalemia has been well documented in numerous randomized controlled trials in the ambulatory setting [20, 32, 33]. Its potential utility in acute hyperkalemia therapy was examined in a multicenter, phase three, double-blind study which randomized 753 patients to receive SZC (either 1.25 g, 2.5 g, 5 g, or 10 g) or placebo three times daily for 48 h [20]. SZC was demonstrated to lower serum K^+^ at 48 h from 5.3 mEq/L to 4.6 mEq/L (*P* < 0.001) while a clinically significant treatment effect was observed at 1 h utilizing the 10 g dose [20]. Kosiborod et al. demonstrated similar clinical significance at 1 h [21].

The ENERGIZE study is the only study to date analyzing SZC in the ER setting. In this randomized placebo-controlled trial (*n* = 70), SZC in addition to insulin and glucose incurred a greater reduction in serum K^+^ at 2 h compared to insulin and glucose alone (−0.72 ± 0.12 vs. -0.36 ± 0.11 mEq/L) [16].

Comparison of the mechanism of action, degree of K^+^ lowering, and potential side effects of the oral potassium binders is summarized in Table 2. As discussed above, data is limited regarding the potential role of the newer potassium binders (patiromer and SZC) for the therapy of acute hyperkalemia. We are addressing this knowledge gap with a head-to-head comparison of the oral potassium binders in the KBindER study.

**Table 2:** Comparison of potassium exchange resins for the treatment of hyperkalemia

**Drug name**	**Sodium polystyrene sulfonate (SPS)**	**Patiromer**	**Sodium zirconium cyclosilicate (SZC)**
Trade name	Kayexalate®, Kionex®	Veltessa®	Lokelma®
FDA approval	1958 (prior to drug regulation laws)	2015	2018
Onset of action	2–24 h	2 h	1 h
Mode of action	The sulfonate molecule exists as sulfonic acid in its ionic state and allows for interaction with a Na^+^ counterion. In this method, Na^+^ is exchanged for K^+^ within the gastrointestinal tract	Utilizes the high colonic K^+^ concentration and exchanges its Ca^2+^ counterion for it. The attached fluorine atom at the alpha-carbon position of patiromer causes electron-withdrawing effects and generates a lower pKa compared to colonic pH. This allows the resin to exist in an ionized state and bind K^+^ effectively	Selectively binds to K^+^ throughout the length of the gastrointestinal tract. SZC mimics physiological K^+^ ion channels by utilizing a thermodynamically favorable binding pocket unique to the size of K^+^
Degree of K^+^ lowering	1.04 mEq/L^a^	0.6 mEq/L^b^	0.4 mEq/L^c^
Location of efficacy	Large intestine	Distal colon	Small intestine and large intestine
Toxicity	Na^+^ loading/fluid overload, hypomagnesemia, hypocalcemia, colonic necrosis	Hypomagnesemia	Edema
Side effects	Nausea, vomiting, diarrhea, constipation, abdominal pain	Mild-severe constipation, abdominal pain	Constipation, abdominal pain
Exchange ion	Na^+^	Ca^2+^	H^+^, Na^+^
Selectivity	Non-specific, K^+^, Ca^2+^, Mg^2+^	Specific, K^+^ (low specificity, Mg^2+^)	Highly specific, K^+^ and ammonium
Resin size	~ 11–124 µm	~ 100 µm	> 3 µm (non-absorbed)
Molecular composition	Polymer resin consisting of sulfonate and attached vinylbenzene (styrene) R group	Polymer resin with attached carboxylic acid and fluorine atom at alpha carbon position	Three-dimensional, seven-member ring lattice made up of alternating zirconium and silicate atoms with adjoining oxygen atoms

^a^1.04 mEq/L mean decrease of serum K^+^ compared to placebo treatment; SPS was given at a dosage of 30 g/day for 7 days [18]

^b^0.6 mEq/L decrease of serum K^+^ 2 h post-treatment with a single dosage of 25.2 g [19]

^c^0.4 mEq/L decrease of serum K^+^ at 2 h post-treatment with dosage of 10 g 3 times daily; clinically significant decrease of K^+^ observed at 1 h [21]

## Methods/design

The KBindER study (K^+^ Binders in Emergency Room and hospitalized patients) is the first trial to compare the efficacy of the 3 oral potassium binders (SPS, patiromer and SZC) in a head-to-head fashion, for the treatment of acute hyperkalemia. We propose a pragmatic study design whereby the cation-exchange resin is added on to standard hyperkalemia treatment per discretion of the treating physician. We have included a mock placebo group with a nonspecific laxative (polyethylene glycol 3350) to investigate the effects of increasing stool output without specific K^+^ exchange, since constipation has been associated with hyperkalemia risk [34].

All study procedures were approved by the University of California, Irvine Institutional Review Board (protocol HS# 2020–5780, approval date August 6, 2020; latest version April 2, 2021). Inclusion and exclusion criteria are summarized in Table 3. Study participants are recruited from adult patients in the ER or main hospital (excluding the intensive care unit) at the University of California-Irvine Medical Center (Orange, CA). Inclusion criteria include plasma K^+^  ≥ 5.5 mEq/L, English or Spanish speaking (consent forms are available in these languages) and able to provide written informed consent. Exclusion criteria include impaired intestinal motility (recent bowel surgery, ileus or bowel obstruction), pregnancy, active psychiatric disorder impairing provision of informed consent, diabetic ketoacidosis, anticipated dialysis treatment within 4 h, or hypersensitivity to any of the study drugs. Patients with pseudohyperkalemia will be excluded: hemolyzed blood samples as reported by the clinical laboratory, severe leukocytosis (white cell count > 50 × 10^9^/L) or severe thrombocytosis (platelet count > 500 × 10^9^/L) [35]. Patients receiving sorbitol are also excluded, given the increased risk of intestinal necrosis if they were to be randomized to receive SPS [23].

**Table 3:** Inclusion and exclusion criteria for the KBindER study

**Inclusion Criteria**	**Exclusion Criteria**
▪ Plasma K^+^ ≥ 5.5 mEq/L ▪ Age ≥ 18 years ▪ English or Spanish speaking ▪ Patient able to provide written informed consent	▪ Recent Bowel Surgery ▪ Ileus or bowel obstruction ▪ Received a dose of an oral potassium binder in past 48 h ▪ Hemolyzed blood specimen as indicated by the laboratory ▪ Severe leukocytosis or thrombocytosis ▪ Pregnancy ▪ Active psychiatric disorder ▪ Diabetic ketoacidosis or hyperkalemia caused by any condition for which a therapy directed against the underlying cause is expected to correct the hyperkalemia ▪ Dialysis session expected within 4 h after randomization ▪ History or hypersensitivity to any of the study drugs ▪ Concurrent use of sorbitol (due to increased risk of intestinal necrosis when used with SPS)

### Study groups

Participants, physicians and house staff are blinded to the study group assignment. Participants undergo sequential randomization to one of 4 study groups:

1. SPS (Kayexalate, Kionex) one dose of 30 g
2. Patiromer (Veltassa) one dose of 25.2 g
3. Sodium zirconium cyclosilicate (Lokelma) one dose of 15 g
4. Polyethylene glycol 3350 (MiraLax) one dose of 17 g (nonspecific osmotic laxative)

Higher doses of the oral potassium binders were chosen to evaluate maximum impact on lowering blood K^+^ with one treatment dose. Given the focus on acute hyperkalemia therapy, it would not have been clinically meaningful to use the lowest starting dose.

### Study procedures

Non-ICU and ER patients are screened remotely by research staff using the Epic Medical Records system to identify potential study candidates. Patients with a qualifying blood K^+^ level (≥ 5.5 mEq/L) are then screened for the inclusion and exclusion criteria (Table 3). Temporizing therapies for acute hyperkalemia (e.g., insulin/dextrose, IV fluids/loop diuretic or albuterol) will be administered per discretion of the attending physician regardless of enrollment status.

A physician listed on the consent form will perform a final criteria review and obtain signed informed consent. These study investigators include hospitalists, ER physicians, and nephrologists. To ensure that the patient and primary treating provider are blinded to the study drug, a nephrologist (WLL) will order the study drug that is dispensed by the Investigational Drug Services Pharmacy. The nurse dispending the study drug is advised not to disclose name of study drug to the participant.

**Fig. 2:**
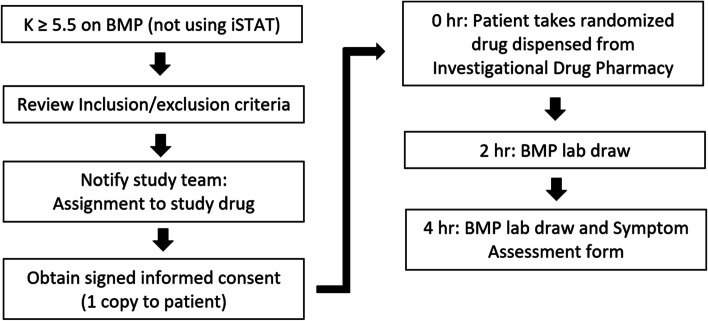
Flowchart of study procedures

Blood chemistries including basic metabolic panel, Mg^2+^ and phosphorus are drawn at 2 and 4 h following administration of the study drug. Participants complete a Symptom Assessment form and report a Palatability Score at 4 h following the medication dose (Supplementary Materials). If the 4-h blood K^+^ remains elevated ≥ 5.5 mEq/L then the primary hospital team will be notified for further hyperkalemia management per their discretion. Data on participant demographics, home and hospital-administered medications, comorbid conditions, lab parameters, and length of ER/hospital stay from chart review will be compiled in a secure REDCap database. Study procedures are summarized in Fig. 2.

An independent Data & Safety Monitoring Board will review potential adverse events every 3 months, or sooner if necessary. This data monitoring committee is comprised of a nephrologist, cardiologist and biostatistician who are not involved in the trial and have no conflict of interest pertaining to the study drugs.

### Study endpoints

Primary endpoints include change in serum K^+^ at 2 and 4 h after the single dose of study medication, admission to hospital (yes/no) for the subgroup of participants enrolled from the ER, and length of ER or hospital stay. The timing of the follow up lab draws is directly communicated to the patient’s nurse. Secondary outcomes of interest include next morning serum K^+^ (for patients remaining in the hospital), need for dialysis within 8 h, change in blood Ca^2+^ and Mg^2+^, and side effects profile. Study endpoints are summarized in Table 4.

**Table 4:** Primary and secondary endpoints

**Primary Endpoints**	**Secondary Endpoints**
▪ Change in serum potassium at 2 and 4 h ▪ Length of ER or hospital stay ▪ Admit to hospital (yes/no) for the subgroup of participants enrolled from the ER	▪ Dialysis yes/no within 8 h ▪ Change in Ca^2+^ and Mg^2+^ ▪ Tolerability (gastrointestinal side effects) ▪ New lower extremity edema ▪ Palatability (patient subjective rating) ▪ Next morning serum potassium

### Sample size calculations

Power analysis was done using conservative estimates from patiromer data (Table 2) [19]. Aiming for 80% power to detect a difference in blood K^+^ lowering of 0.6 mEq/L between the nonspecific laxative and oral K^+^ binder, within-group standard deviation 0.5 mEq/L, with alpha error 0.01 (to adjust for multiple comparisons due to multiple randomization groups), at least 18 subjects per group are needed to detect a significant difference between the groups. We are aiming for 20 patients per group for final analysis to ensure adequate numbers for comparison and account for potential missing data (i.e., participants who do not complete the 2 timed lab draws). Our study is not intended to identify superiority of one oral K^+^ binder vs other binders.

### Statistical analysis

Baseline characteristics between the four groups will be compared using Chi-square test for categorical variables or ANOVA with contrasts and Kruskal-Wallis test (non-parametric) for continuous variables. Variables of interest include acute kidney injury, CKD or chronic dialysis status; diabetes mellitus; congestive heart failure; RAAS inhibitor medication prescription; metabolic acidosis; and concurrent therapies for hyperkalemia (insulin/dextrose, diuretics, albuterol, bicarbonate).

Linear mixed effects models will be used to compare mean change in blood K^+^ from baseline between the four groups at 2 and 4 h, and next morning K^+^ (P set at 0.05). Multivariate adjustment will be done for kidney function, diabetes mellitus, congestive heart failure, metabolic acidosis, RAAS inhibitor prescription, and treatment with temporizing agents (insulin, albuterol, loop diuretics). For next morning K^+^ values, multivariate adjustment will include any hyperkalemia treatments that were done after the 4-h study protocol (including dialysis). A Cox proportional hazards model will be used to analyze the association between type of oral potassium binder and length of ER or hospital stay.

Safety and tolerability will be assessed using a Kruskal-Wallis test to globally test for a negative trend across all 4 study treatments (two-sided *P* < 0.05) and, if significant, further testing will be done using a two-sided Fisher Exact test compare each potassium binder vs. the nonspecific laxative control.

## Discussion

To our knowledge, the KBindER trial is the first to evaluate the efficacy of SPS, patiromer and sodium zirconium cyclosilicate head-to-head in ER and hospitalized patients with acute hyperkalemia. The results will be highly relevant to patient outcomes and healthcare spending; patients who are admitted to the hospital with hyperkalemia spend on average 4 additional days in the hospital compared to matched, non-hyperkalemic inpatients and incur USD $15,606 higher in total health care costs over a one year period [4]. This study will be indirectly assessing health care costs by identifying if there is a difference in hospital length of stay amongst the K^+^ binders.

Based on available data (Table 1), we hypothesize that SZC will have the most rapid onset of action to decrease blood K^+^ via gastrointestinal excretion, if used as stand-alone therapy for acute hyperkalemia. However, it is important to test the oral potassium binders in the real-world setting where temporizing agents are commonly given to shift K^+^ intracellularly, to determine the degree of additional K^+^ lowering with the potassium binder. Further, we will evaluate whether patients treated with a potassium binder are more likely to maintain normokalemia on next-morning labs (for patients remaining in the hospital).

It is timely to re-evaluate the utility of SPS in the treatment of acute hyperkalemia. If SPS proves to have equivalent or lower efficacy in lowering K^+^ this may justify the higher cost of the newer oral binders (patiromer, SZC) to avoid the SPS-associated risk of colonic necrosis. Significant side effects such as hypercalcemia (patiromer) or edema (SZC) are not expected with a single dose of oral potassium binder. The oral K^+^ binders offer a favorable safety profile compared to some widely-used temporizing measures for hyperkalemia. For example, insulin can incur life-threatening hypoglycemia despite co-administration of glucose [11, 16], particularly in CKD patients who have reduced renal clearance of infused insulin and compromised gluconeogenic pathways [36]. In a retrospective analysis of late-stage CKD patients being treated with insulin (*n* = 221), 13% of patients developed hypoglycemia [36]. The use of i.v. bicarbonate in patients with metabolic acidosis is controversial due to the concern of displacing hydrogen ions from albumin, thus freeing up binding spots on albumin for Ca^2+^ and lowering ionized Ca^2+^. Further, i.v. bicarbonate can itself directly bind Ca^2+^ thus causing hypocalcemia [37] and incurring risk of myocardial destabilization.

Our study is not intended to identify superiority of one oral K^+^ binder vs other binders, and we acknowledge the limitation that differential efficacy of the oral K^+^ binders may be under-studied if the actual group difference is less than 0.6 mEq/L.

In summary, the KBindER trial is the first head-to-head evaluation of oral potassium binders in a real-world setting where temporizing therapies will be given per discretion of the medical team. The findings from our study will inform decision-making guidelines for the treatment of acute hyperkalemia.

## Supplementary Information

**Additional file 1.** 4-hour symptom survey form.

## Data Availability

The de-identified dataset from the current study will be available as a supplementary file with the manuscript, and will be available immediately following publication. De-identified study findings will be reported on ClinicalTrials.gov.

## Abbreviations

CKD: Chronic kidney disease
ER: Emergency Room
ICU: Intensive care unit
K^+^: Potassium
Na^+^: Sodium
RAAS: Renin-angiotensin-aldosterone system
SPS: Sodium polystyrene sulfonate
SZC: Sodium zirconium cyclosilicate
