# Supplementary material for: A randomized study to compare oral potassium binders in the treatment of acute hyperkalemia
Source: BMC Nephrol. 2023 Apr 5;24:89. doi: 10.1186/s12882-023-03145-x (PMC10074796; doi:10.1186/s12882-023-03145-x)
Supplement: Supplementary file 1 — Additional file 1. 4-hour symptom survey form. [file 12882_2023_3145_MOESM1_ESM.docx]

*Supplemental Materials*

**Subject Symptom Assessment**

**Subject’s first and last name (print):** ___________________________________________________

**Date this form was completed:** __________________________________________________________

**Was this form completed by someone other than the subject? □ No □ Yes**

**If Yes, record person’s name here:** __________________________________________

| Yes | No | Sign/Symptom | If Yes, provide a brief description of the event |
| --- | --- | --- | --- |
| **□** | **□** | Nausea |  |
| **□** | **□** | Vomiting |  |
| **□** | **□** | Diarrhea |  |
| **□** | **□** | Constipation |  |
| **□** | **□** | Flatulence/bloating |  |
| **□** | **□** | Abdominal pain |  |
| **□** | **□** | Leg swelling |  |
| **□** | **□** | Palpitations |  |
| **□** | **□** | Any other symptoms |  |

**Palatability score** (circle one)

Really bad Ok Really good

1 2 3 4 5
